# Eosinophilic Esophagitis: An Emerging Disease in Childhood – Review of Diagnostic and Management Strategies

**DOI:** 10.3389/fped.2014.00129

**Published:** 2014-11-21

**Authors:** Alexandra Papadopoulou, Jorge Amil Dias

**Affiliations:** ^1^First Department of Pediatrics, Athens Children’s Hospital “Agia Sofia”, University of Athens, Athens, Greece; ^2^Department of Pediatrics, Hospital S. João, Porto, Portugal

**Keywords:** eosinophilic esophagitis, oral viscous budesonide, fluticasone propionate, oral steroids, amino acid-based formula, empiric elimination diet, targeted elimination diet

## Abstract

Eosinophilic esophagitis is a chronic immune/antigen mediated inflammatory disease of the esophagus. It comprises a separate entity of increasing incidence and prevalence in children and adults. The disease is characterized by histological evidence of dense esophageal tissue eosinophilia in the presence of a variety of upper GI symptoms including vomiting, dysphagia, food impaction, and odynophagia. Cornerstone of treatment is dietary intervention and/or the off-label use of swallowed topical corticosteroids. New drug therapies are under investigation. In this review, we focus on the diagnostic approach and the currently available treatment strategies.

## Introduction

Eosinophilic esophagitis (EoE) is a chronic immune/antigen mediated esophageal inflammatory disease associated with esophageal dysfunction, resulting from severe eosinophil-predominant inflammation (1). The prevalence of the disease varies from 0.89/10,000 in Western Australia (2) to 4/10,000 children in Ohio (3) while, in Europe, the incidence of the disease was reported to be 0.16/10,000 in Southern Denmark (4). A recent paper reveals that incidence and prevalence has increased considerably throughout the world (5). Exact epidemiologic figures depend on availability of endoscopy services, medical awareness, and diagnostic protocol.

Eosinophilic esophagitis is a disease with several phenotypes [e.g., structuring/gastro esophageal reflux disease (GERD)-like/dysmotility], which need to be better defined in order to clarify long-term complications such as the development of fibrosis. The disease is more common in males and in patients with atopic diseases (6). Studies conducted in children suggest that in many patients, symptoms of EoE are triggered by food allergens (1). Experimental models suggest that other sources of antigen exposure beyond food may also cause EoE (7) and a recent report describes three adults developing EoE after clearly identified exposure to aeroallergens (8). Whether this occurs also in pediatric patients remains to be demonstrated, although seasonal exacerbation of the disease has been reported in children with EoE (9). The elimination of specific foods from the patient’s diet is associated with disease remission while, their reintroduction induces relapse. However, the methodology for identification of potentially significant food- or aero-antigens requires further development as the currently available allergy tests often give false positive or false negative results leading to the incomplete elimination of causative food allergens from the patient’s diet and to inability to resolve symptoms and histological abnormalities. The first consensus recommendations for diagnosis and treatment of EoE were published in 2007 by a group of experts who updated them in 2011 (1) while, more recent guidelines were published by American College of Gastroenterology (10), and the ESPGHAN (11). The latter, provided practical management guidelines of childhood EoE based on evidence where available and on expert opinion where evidence was lacking, and also, practical diagnostic and management algorithms to guide pediatric gastroenterologists in clinical practice. In the present review, we discuss diagnosis and treatment options of childhood EoE.

## Clinical Manifestations of EoE and Diagnostic Approach

The clinical manifestations of EoE are variable depending on age and the disease phenotypes. Feeding difficulties are the most common symptoms in infants and toddlers, vomiting and pain in children, and dysphagia and food impaction in adolescents. Patients with EoE may or may not be atopic. Total IgE and specific IgE to food antigens (RAST tests) are not reliable for the identification of causative foods of EoE. Skin prick tests (SPT) and allergen patch tests (APT) can be used but the latter need validation and are not available everywhere. The foods that are considered for testing with skin SPT and APT tests include milk protein, egg, peanuts, soy, a variety of grains (wheat, rice, corn, rye, oats, and barley), meats (beef, pork, chicken, and turkey), fish, and shellfish. The positive predictive values of SPTs in children with EoE were reported to range between 26 and 86% (highest for milk) while the negative predictive values ranged between 29 and 99% (highest for peanut) (6). The sensitivity and specificity of the tests varied between 18–88 and 82–97%, respectively (6). Therefore, isolated SPTs may have a better value to exclude rather to confirm relation to specific foods. The combination of SPTs and APT tests increased the negative predictive value to an average of 92% with the exception of milk (at 44%), while the positive predictive value remained low (at 44%) (6). As the most common food triggers of EoE the following have been recognized: milk (55%), wheat (33%), nuts (33%), and seafood (11%) in adults (12) with EoE while, in children (6), milk was the most common food identified, followed by wheat, soy, and eggs (6). The use of allergy tests is limited by common false positive and false negative results. The identification of food allergens in patients with EoE may mean concomitant food allergy without those foods being the precipitating cause of the disease. On the other hand, elimination diets may still contain the offending product in occult form leading to refractoriness to the elimination diet.

**Figure 1:**
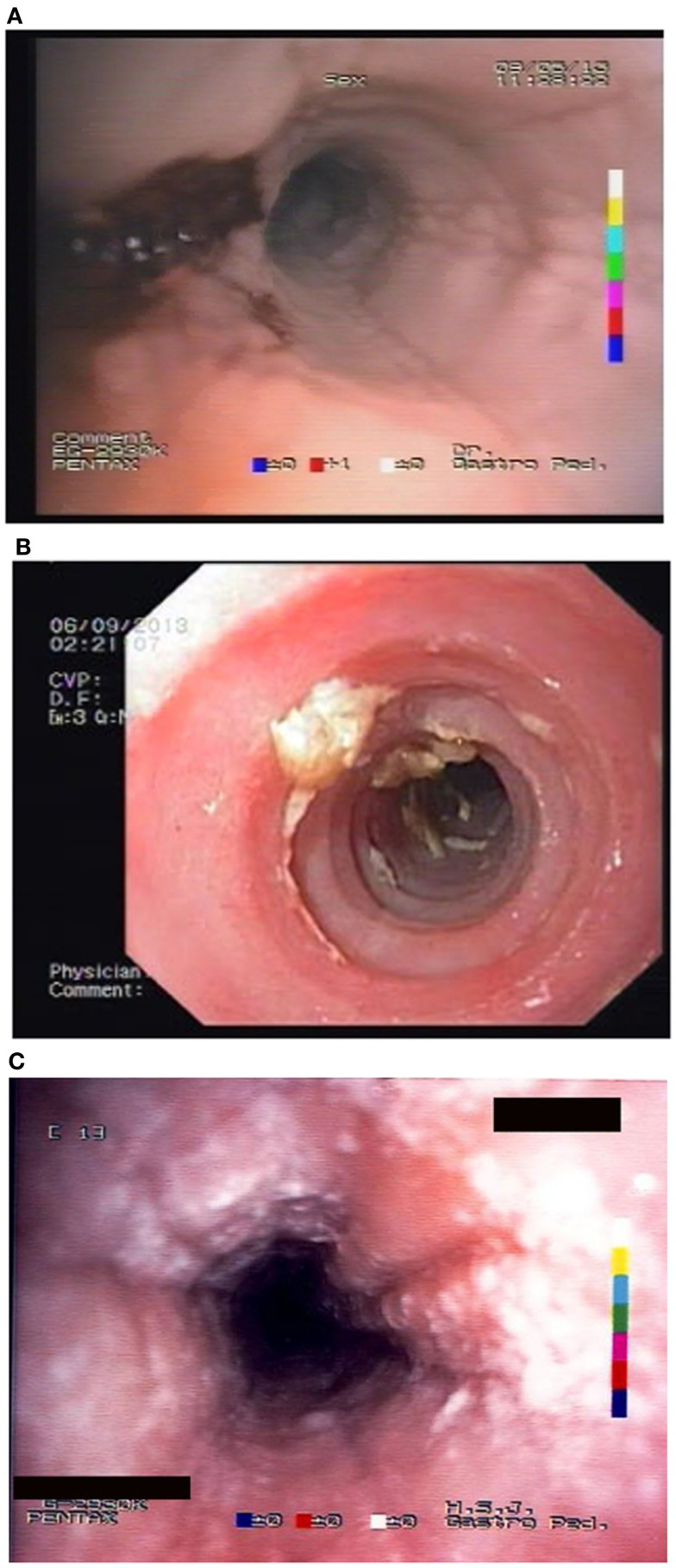
**Endoscopic appearance of EoE showing (A) friability of the mucosa and easy bleeding; (B) trachealisation of esophagus with remains of recent food impaction; and (C) edema with furrows and white spots of eosinophil granulomas**.

**Figure 2:**
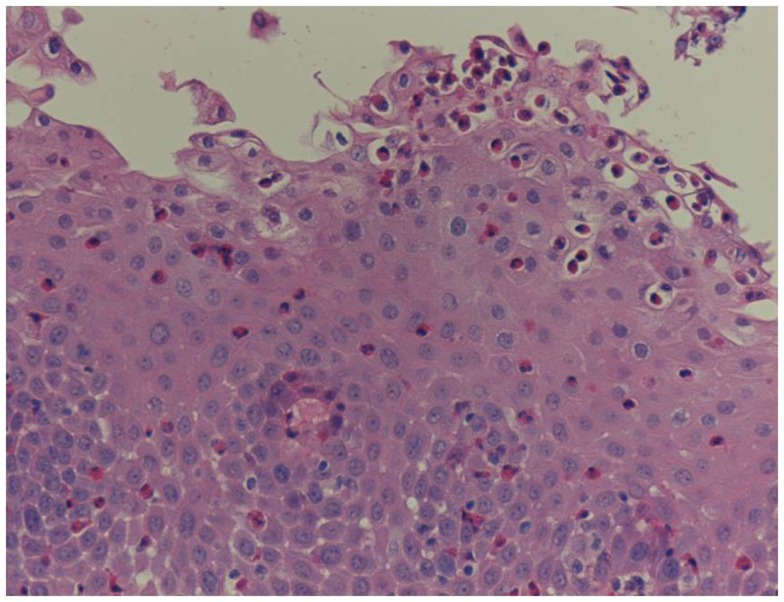
**Histology of EoE with marked infiltration of eosinophils in the mucosa**.

Unfortunately, there are no available specific biomarkers for the diagnosis of the disease, the monitoring of the response to treatment, and the disease prognosis. Therefore, the disease diagnosis relies currently only on endoscopy and histology (3). The endoscopic features of the disease vary from normal esophagus to the presence of esophageal rings, furrows, and/or white exudates (Figures 1A–C) indicative of eosinophilic microabscesses and less often narrowing of the caliber of esophagus (13). The presence of mucosal breaks (erosions or ulceration) are not indicative of EoE but of GERD, Crohn’s disease, or other diagnoses. The main histologic feature of EoE is striking eosinophilia of esophageal mucosa, usually along with microabscesses, superficial layering, or extracellular eosinophil granules (Figure 2). The presence of at least 15 eosinophils per high-power field found in at least 1 esophageal mucosal biopsy (peak value) is required for the histological definition of the disease. At least three esophageal biopsies are needed from different parts of esophagus to achieve a diagnosis of EoE in 97% of patients (14) and five, to achieve a diagnosis of EoE in 100% (15). The need for multiple biopsies derives from the fact that eosinophils are recruited from the deeper layers of the esophageal wall and areas with lower eosinophil density may exist in endoscopic superficial mucosal biopsies (16). If we rely, therefore, on a limited number of superficial mucosal biopsies, the diagnosis of EoE may be missed. Furthermore, it should be noted that esophageal eosinophilia is not an exclusive feature of EoE. Other diseases that are associated with esophageal eosinophilia are GERD, Crohn’s disease, connective tissue disease, infectious esophagitis (herpes, *Candida*), celiac disease, achalasia, graft-versus-host disease, drug hypersensitivity, eosinophilic gastroenteritis, and hyper eosinophilic syndrome (1, 17). Gastric and duodenal biopsies should also be taken on first diagnostic endoscopy to identify or exclude other conditions like eosinophilic gastroenteropathy. The discussion of this poorly characterized disease is beyond the scope of this paper. GERD is the main differential diagnosis from EoE. GERD can present with similar symptoms as EoE or even co-exist with EoE. There are studies proposing scoring systems to differentiate between GERD and EoE, based on clinical and endoscopic features: male gender, dysphagia, history of food impaction, absence of pain/heartburn, linear furrowing, and white papules (18). Such systems may be useful in older children and in adolescents (18). The identification of mucosal inflammatory mediators related to eosinophil activation has also been tested and may give new diagnostic options and accuracy (19). Until more data become available, however, there is a wide consensus that patients need endoscopic and histologic assessment after a course of 2 months trial with antisecretory drugs (proton-pump inhibitors, PPIs). Those patients with esophageal eosinophilia who improve both clinically and histologically after the treatment with PPIs are currently classified as having either GERD or PPI-responsive esophageal eosinophilia (PPI-REE). Further studies are required to show whether PPI-REE is a separate entity or it comprises a subtype of EoE or GERD. The ability of PPIs to achieve resolution of esophageal eosinophilia is attributed to acid-suppression but also to possible inhibition of various other inflammatory mechanisms (1, 20, 21). The lack of response to high-dose proton-pump inhibitors is necessary to fulfill the current definition criteria for the disease (1, 11). The recommended dose of PPIs is 1 mg/kg per dose, twice daily with maximum dose reaching adult dose 20–40 mg once or twice daily depending on patient and PPI. After the confirmation of the diagnosis of EoE, PPIs are usually stopped unless there is evidence of coexisting GERD. In that case, PPIs may be continued as adjunctive therapy to other specific for EoE interventions (1). A practical algorithm on the diagnostic approach of children and adolescents with symptoms suggesting EoE is given in the position paper recently published by ESPGHAN (11).

## Management of EoE

The goals of treatment of EoE are to achieve and maintain clinical and histological remission of the disease and to prevent iatrogenic damage such as nutritional compromise due to long-term elimination diet. Until minimal invasive tests measuring biochemical products of eosinophil activation are available to facilitate adequate monitoring of the inflammatory process, the confirmation of histological remission of the disease with repeat endoscopy and biopsies before food reintroduction or drug titration, is necessary.

Dietary elimination and/or the off-label use of topical corticosteroids is usually associated with reversal of symptoms and histological abnormalities of the esophagus. A practical algorithm to treatment approach is given in the position paper recently published by ESPGHAN (11). It should be noted, however, that the optimal intervention needs to be individualized as atopic patients most likely benefit from the elimination diet and the non-atopic from steroids. Unfortunately, it is not always easy to identify the causative foods while, not all of the atopic patients are sensitized to the same foods and others are sensitized to aero allergens.

## Dietary Treatment

Three elimination diets have been developed for patients with EoE: (1) amino acid-based formula (AAF); (2) targeted elimination diet (TED); and (3) empiric elimination diet (EED).

Case series suggest all of the above diets are effective in inducing clinical and histological remission in patients with EoE (6, 22–28) with AAF being the most successful (Table 1), It should be noted, however, that, to date, there are no randomized controlled trials investigating the efficacy of any of these diets. There also has not been any head-to-head comparison of these different modes of treatment and different centers have developed varying levels of expertise in administration of them, which is likely to influence management preference.

**Table 1:** **Outcomes of studies on dietary treatment of eosinophilic esophagitis**.

Reference	No of patients	Diet	Duration	Outcome
Kelly et al. (23)	10 Children	AAF	Min. 6 weeks	Significant clinical and histology improvement in all
Markowitz et al. (22)	51 Children	AAF	4 weeks	Significant improvement in all
Henderson et al (24)	90 Children	AAF 49	Aver. 18 weeks	Histologic remission in 96%
		SFED 26	Aver. 18 weeks	Histologic remission in 81%
		TED 15	Aver. 16 weeks	Histologic remission in 63%
Kagalwalla et al. (27)	60 Children	AAF 25	Min. 6 weeks	Significant improvement AAF 88%
		SFED 35		Significant improvement SFED 74%
Gonsalves et al. (25)	50 Adults	SFED	6 weeks	Significant clinical improvement in 94%
				Histological improvement in 70%
Spergel et al. (30)	146 Children	TED	4–8 weeks	Significant improvement 77%
				Partial improvement 13%
				Treatment failure 10%
Teitelbaum et al. (26)	11 Children	TED	4–6 weeks	No improvement

The AAF consists of complete removal of food allergens from the diet substituted by a hypoallergenic formula based on amino acids (23). Several studies reported efficacy of AAF in achieving clinical and histological remission of EoE in both children (22, 24) and in adults (28). Resolution of clinical symptoms such as vomiting, abdominal pain, or dysphagia in children (22, 24) and dysphagia, chest pain, food impaction, or heartburn in adults (28), was reported as early as at 8 days (22) or at 2 weeks (24, 28) of introduction while, histological resolution was reported at 4 weeks (22, 24, 28). Despite these encouraging reports of remission induction, long-term use of the AAF is severely curtailed by its many disadvantages including the high cost of AAF and the frequent need for nasogastric tube placement or even gastrostomy due to poor long-term palatability. Currently, AAF is reserved for young infants with multiple food allergies as well as for patients who do not respond or do not wish to follow strict diet with multiple food elimination.

Owing to the poor long-term acceptability of AAF, the TED, which removes foods based on a combination of suggestive history of food triggers and results of SPTs and (in some centers) APT was evaluated. The benefit of TED to induce remission in children has been variable from non- to moderate (29) or high (6) among those with positive SPTs and/or APT. It should be noted, however, that some patients with EoE have intolerance to multiple food antigens some of which may have not been identified by skin tests. Furthermore, the identified food antigens with SPTs and/or APT may not necessarily be the causative foods of the disease. Therefore, relying only on tests, which have often false positive or false negative results may lead to elimination from the diet of only part of the offending food antigens, and failure to induce remission.

Another dietary strategy for treating EoE is with the use of EED, which removes from the diet independently of sensitization, the known allergens that strongly correlate with EoE, which are often the dairy products, soy, eggs, wheat, peanuts, fish, and shellfish (27). This diet strategy has been used in treating children with EoE achieving clinical and histological improvement in 74% of patients (27), although a more recent study reported a lower percentage of the patients showing histological remission (53% of patients), which was attributed by the authors to the poor dietary compliance (6).

The performance of diet treatment requires supervision by an experienced dietitian to ensure compliance with the diet and that proper amount of calories, vitamins, and micronutrients are maintained (11). Nutritional status of the patient needs to be evaluated longitudinally in order to identify early nutritional impairment and apply appropriate measures to reverse it (11).

### How to assess the efficacy of the diet?

Histological response does not correlate with clinical response. Relying on symptom reversal for the assessment of the efficacy of a therapeutic intervention may be misleading, allowing perpetuation of esophageal inflammation. The recommendation, therefore, is to assess the efficacy of the chosen treatment with a repeat endoscopy following resolution of symptoms after commencing dietary elimination (11). In case AAF is chosen, the repeat endoscopy may be performed at 4 weeks as the resolution of symptoms is achieved earlier. In case of TED or EED, the resolution of symptoms is expected later and therefore the repeat endoscopy is suggested at 8–12 weeks from the introduction. In case of histological remission, food reintroduction is considered starting from the least allergenic foods (30). During food reintroduction, those foods that prove to trigger EoE symptoms may need to be indefinitely restricted (1). Some units advise the invasive approach of performing periodic endoscopies to assure maintenance of combined symptomatic and histological remission following food reintroduction and suggest a re-endoscopy after reintroduction of all of the foods of similar allergenicity from vegetables, fruits, grains, and meat. More studies are required to show whether the measurement of specific inflammatory mediators through non-invasive techniques may allow an easier monitoring of the tolerance to specific foods.

As stated above, dietary treatment is particularly effective in treating EoE in atopic children. Patients without a history of atopy are relatively refractory to dietary treatment and require initiation of drug therapy (31). In some patients, there is evidence that seasonal exacerbations caused by inhaled aeroallergens (including pollens and molds) may occur, often characterized by food bolus impaction (32). It is, therefore, important to enquire about seasonal exacerbations and, if present, to try to identify triggering aeroallergens. In case of an established pattern of seasonal exacerbations, preventive measures with dietary restrictions and/or the use of topical corticosteroids may be suggested.

## Drug Therapy

Among the medications that have been assessed in pediatric patients with EoE with different success, are corticosteroids (oral systemic and topical), cromolyn sodium, leukotriene receptor antagonists, and biologics (mainly anti-IgE and anti-IL-5 monoclonal antibodies). From those, only oral systemic and topical corticosteroids proved to be highly effective in treating children with EoE (Table 2). It should be noted, however, that there are only few randomized controlled trials assessing the efficacy of different drug agents, therefore, further studies are needed to have a universal approach to EoE treatment.

**Table 2:** **Outcomes of studies on steroid therapy of eosinophilic esophagitis**.

Reference	No of patients	Treatment	Duration	Outcome
Liacouras et al. (33)	20 Children	Oral steroids	4 weeks	Clinical and histological response in all
Teitelbaum et al. (26)	11 Children	Topical FP open label	8 weeks	Clinical and histological response in all
Remedios et al. (35)	19 Adults	Topical FP open label	4 weeks	Clinical and histological response in all
Konikoff et al. (36)	36 Children	Topical FP randomised controlled trial	12 weeks	Clinical improvement
				FP: in 67%
				Placebo: in 27%
				Histological remission
				FP: in 50%
				Placebo: in 9%
Dohil et al. (38)	24 Children	Topical OVB DBCT	12 weeks	Clinical improvement
				OVB: in 87%
				Placebo: in none
				Histological improvement OVB: in 87%
				Placebo: in 0%
Straumann et al. (39)	36 Adolescents and adults	Topical OVB DBCT	2 weeks	Clinical improvement
				OVB: in 72%
				Placebo: in 22% histological improvement following OVB but not following placebo

### Topical and systemic oral steroids

Oral systemic and topical steroids are both highly effective in inducing clinical and histological remission in adults and in children with EoE with minor side effects such as oral candidiasis, resolving following drug discontinuation. The clinical remission following oral steroids is achieved as early as at 1 week from the start of treatment and the resolution of histologic lesions at 4 weeks (33). Unfortunately, the discontinuation of drug therapy is often associated with relapse of the disease and the need for a repeated course of treatment. Considering, therefore, the risks associated with the chronic use of oral systemic steroids in children, topical steroids were assessed in patients with EoE and proved effective in achieving resolution of histologic lesions. Studies showed that although oral prednisone achieved a greater degree of histologic regression, there was no statistical difference with regards to symptom resolution, symptom relapse, or time of relapse (34). The use of systemic corticosteroids, therefore, is only considered when immediate relief of the patient’s symptoms such as severe dysphagia, dehydration, weight loss, or esophageal strictures, is needed. In all other case, the topical steroids are considered as first line drug treatment for EoE. The effective dose for eliminating clinical symptoms and histologic abnormalities is 1–2 mg/kg/day of prednisone with maximum dose reaching 40–60 mg.

The topical steroids, which have been used in treating EoE, are swallowed fluticasone propionate and oral viscous budesonide (OVB). Fluticasone propionate is sprayed into the mouth with lips sealed around the device and the patient is advised to not drink or eat for the next 30 min (35). This drug was effective in both adults (35) and children (26) with EoE and was reported to induce remission in 50 (36) to 91% of the patients (37). The suggested dosage ranges from 88 to 440 μg twice to four times daily for children and 440–880 μg twice daily for adolescents/adults (1).

Oral viscous budesonide is also an option for treating EoE. It is prepared by mixing liquid solution of budesonide 1 mg/2 ml (the preparation used for inhalations) and 5 g of sucralose. The administration of this preparation achieves regression of symptoms and of endoscopic and histologic abnormalities in 87% of children (38) and in 72% of adolescents and adults with EoE (39). The recommended dosage of OVB is 1 mg daily for children <10 years and 2 mg daily for older children and adults (1). Both topical preparations may induce remission of inflammation as documented in various studies but the OVB may provide increased concentration of the drug in the esophagus (40).

Drug titration should be initiated after confirming histologic remission following symptoms resolution with a repeat endoscopy, at 4–12 weeks following drug introduction (11).

It should be noted, however, that similarly to oral systemic corticosteroids, the discontinuation of topical steroids is associated with relapse of symptoms as early as at 4 months (38) or according to others at a mean time of 8.8 months (37) requiring maintenance therapy. In adults with EoE, a low-dose (twice daily 0.25 mg) of OVB maintained quiescent EoE in remission (41). In children, although the need for maintenance treatment is often recognized, the optimal regimen still needs to be determined.

### Other drug therapies

Other drug therapies such as sodium cromoglycate or montelukast, a leukotriene receptor antagonist, are not recommended for treating EoE unless more favorable data become available (1, 11). The same is true for immunomodulating drugs and biologics (42). The efficacy of antibodies against IL-5 in patients with EoE is controversial showing variable results and relapse upon discontinuation, while anti-IgE monoclonal antibodies were effective in improving food tolerance and reversing symptoms but not in improving endoscopic and histological abnormalities (43).

## Follow-Up of Asymptomatic Patients

The follow-up of asymptomatic patients is not universal and differs widely among centers with some performing periodic endoscopic re-evaluations while, others not. Considering that long-term complications of the asymptomatic disease are poorly defined, the follow-up of asymptomatic patients should be individualized considering disease phenotype and severity (11).

As mentioned above, there may be a discrepancy between symptoms and histological features. Medical advice may be therefore guided to promote inflammation-free esophageal mucosa with follow-up endoscopies, but this ambitious goal has to be adjusted to individual patients.

## Esophageal Dilatation

Esophageal dilatation (ED) has been used mainly in adults with EoE (44). ED can be helpful in acutely symptomatic patients who present with severe dysphagia due to marked esophageal narrowing after failure of medical treatment to improve symptoms (45). It should be stressed, however, that before deciding on ED, it is mandatory to try medical and/or dietary therapy (11).

## Conclusion

Eosinophilic esophagitis is a chronic, relapsing inflammatory disease of the esophagus, which requires often repeated or prolonged therapy. The definition of the disease phenotypes, and the development of biomarkers, to evaluate the response to treatment and the early relapse, will allow guide more precisely short- and long-term management of the disease.

## Conflict of Interest Statement

The authors declare that the research was conducted in the absence of any commercial or financial relationships that could be construed as a potential conflict of interest.

## Appendix

### Key points

A trial with antisecretory medication is necessary to exclude GERD and PPI-responsive esophageal eosinophilia and to fulfill the diagnostic criteria of EoE.

Elimination diet and/or off-label use of topical corticosteroids are effective measures for treating EoE.

Elimination diet is the first line treatment in atopic children.

Systemic corticosteroids are reserved for patients with severe disease requiring immediate relief, or when other treatments have failed.

Cromolyn sodium (sodium cromoglycate) and leukotriene receptor antagonists are not currently recommended for treating EoE due to lack of solid evidence of benefit.

Immunosuppressive drugs and biologics have shown some value but effect has been limited and therefore not yet recommended as standard therapy.
